# Intervention Synthesis: A Missing Link between a Systematic Review and Practical Treatment(s)

**DOI:** 10.1371/journal.pmed.1001690

**Published:** 2014-08-12

**Authors:** Paul P. Glasziou, Iain Chalmers, Sally Green, Susan Michie

**Affiliations:** 1 Centre for Research in Evidence-Based Practice, Faculty of Health Sciences and Medicine, Bond University, Gold Coast, Queensland, Australia; 2 James Lind Initiative, Oxford, United Kingdom; 3 Australasian Cochrane Centre, Monash University, Melbourne, Victoria, Australia; 4 Centre for Outcomes Research and Effectiveness, Department of Clinical, Educational and Health Psychology, University College London, London, United Kingdom

## Abstract

Paul Glasziou and colleagues discuss methods to guide selection of an intervention from individual trials within a systematic review.

*Please see later in the article for the Editors' Summary*

**Summary Points**

- Effective delivery of treatments requires clear procedural details of the essential elements of treatment. Hence, if a systematic review finds that a class of interventions is effective, then the users of the review will want to know: “Which version of the intervention should I use?”
- Current methods to guide selection or synthesis from the variations of a treatment used across trials in a systematic review are poorly developed, and absent from most instructions on systematic review methods.
- We identify three basic approaches: (i) single-trial-based choice, where criteria such as feasibility, cost, effectiveness, or familiarity guide which trial's treatment to adopt; (ii) common components hybrid, which extracts then combines—based on frequency and importance—components of several trials; and (iii) model-guided synthesis, where a model of the mechanisms of effect is used to code and assess the importance of components for the version(s) recommended.
- Whichever method is used, we suggest review authors provide an “intervention options table”, which describes the pros and cons of some intervention alternatives used in an individual trial or set of trials.
- If clinicians and policymakers are to be expected to base their practices on the results of systematic reviews in practice, these three approaches will need to be more widely adopted.

## Background

Effective delivery of treatments requires clear procedural details of the essential elements of treatment. Hence the CONSORT statement requests that trial reports provide “the interventions for each group with sufficient details to allow replication, including how and when they were actually administered”. This detail is often lacking, however, and systematic reviews of trials are further complicated by variations in interventions. If a systematic review finds that a class of interventions is effective, then the users of the review will want to know: “Which version of the intervention should I use?” Systematic reviews will usually examine several trials with closely related, but rarely identical, interventions: the details of the interventions will vary across trials. Even for relatively simple clinical interventions, such as prescribing antibiotics for acute sinusitis, the specific antibiotic, dose, duration, and possibly frequency may vary. For more complex interventions, such as strategies to implement clinical practice guidelines, heterogeneity of intervention content and mode of delivery are likely to be greater [1]. For example, a review of exercise for patients with knee osteoarthritis found that it reduced pain and improved function [2], but the studies used different types and doses of exercise. A subsequent meta-analysis found that the best programmes focused on quadriceps muscle strength and had supervised exercise at least three times per week [3], which would help guide the choice of exercise programme. However, to be confident that the implementation of the findings in practice is likely to be effective, the user also needs to know the types and intensity of exercise(s), the duration of sessions, the schedule, processes of tailoring or adjustment, and the context (for example, physical setting and social influences) and modes of delivery (for example, patient handouts or other materials needed by patients trying to implement the intervention).

Whilst this can be a problem even in reports of individual trials [4],[5], having an adequate description of the intervention to be used poses an even greater problem in reports of systematic reviews [6]. A review of 80 studies selected for summarizing in the journal *Evidence-Based Medicine* because they were both valid and important for clinical practice found that the intervention could be replicated by clinicians in only half of the reports selected, and that this proportion was much lower for interventions recommended in reports of systematic reviews than for interventions recommended in individual studies [7]. The problem is partly due to poor reporting (and complexity) of the interventions within the included trials, but a further complication results from variations in interventions across trials.

Current methods to guide the translation of evidence in systematic reviews to the selection and prescription of a specific intervention within a class of interventions are poorly developed and ignored by most textbooks on systematic reviews and intervention design. The three basic approaches are to (i) select the intervention used in an individual trial; (ii) combine components of several trials—thereby creating a new, “synthetic” composite version of the intervention; or (iii) pick an intervention or create a composite version guided by a model of the mechanisms of the effect. Whichever method is used, we suggest review authors provide an “intervention options table”, which describes the pros and cons of some intervention alternatives used in an individual trial or derived from set of trials.

Choosing an intervention from a single trial—option (i)—is simplest: it is more direct and requires little additional work, and it avoids assumptions about the untested effects of a composite intervention. However, the systematic review evidence of effect is based not on any single trial, but on a synthesis of findings. Basing the intervention on components from several, or all, trials—option (ii)—has the appeal of using the components judged to be the best from all the interventions—provided “the best” can be identified. While attractive, this approach implies considerable additional work in describing, comparing, and analysing the included trials, to identify the potentially active components that are often insufficiently well described [8]. Because of the heterogeneity of interventions, there will only rarely be the statistical power to detect whether or not components have contributed to the observed effects of interventions. Guiding a synthesis by an understanding of the intervention's mechanisms—option (iii)—requires a theoretical understanding that may not have guided the review or that may not be accepted by the practitioners wishing to implement the review evidence.

This article considers possible methods for proceeding from the evidence in systematic reviews to a choice of specific interventions (medical treatments, public health interventions, health service interventions, etc.). We have searched the published literature to identify methods for addressing this challenge, checked the references of papers describing approaches and methods, and conducted a forward citation search from relevant articles identified. Our article is a synthesis of existing and some new methods, and describes the three basic approaches and the advantages and limitations of each.

## Options for Specifying Potential “Best Bets” among Interventions Considered in Systematic Reviews

Though there is some overlap between the methods proposed, the methods can be separated into three basic approaches: single-trial-based choice, where an intervention category, and a specific version of it, is selected based on several criteria such as the effect size, practicality, cost, and relevance to a particular setting, from among the tested interventions; common components hybrid, which is a recombination approach where a composite (new) intervention is constructed from the components of the interventions tested in some of the studies included in the systematic review; and model-guided synthesis, where the choice of a single or combined intervention is guided by a theory of how the interventions achieve their effects. Table 1 sets out some details and differences of these three basic approaches.

**Table 1:** Definitions and methods of the three basic approaches for intervention synthesis.

Attribute	Single-Trial-Based Choice	Common Components Hybrid	Model-Guided Synthesis
Description	Pick (or rank) the “best” intervention(s) from those used across all trials	Develop a composite intervention based on components of the interventions in all trials	Analyse interventions guided by a model of the mechanisms of action
Processes	Establish decision criteria, who is going to do the ranking, and how consensus is to be achieved	List all components, code components from trials, and select common components	Propose mechanism, code trial interventions, and conduct subgroup analyses or meta-regression
Output	Ranked trial interventions, consensus data, and selected single intervention	Composite intervention derived from all interventions	A single study intervention or a composite
Assumptions and requirements	Minimal assumption: at least some interventions replicable; requires agreement about criteria for “best”	Requires that sufficient details of interventions can be obtained	Requires that sufficient details of interventions can be obtained, and sufficient diversity of studies to allow analysis such as meta-regression
Limitations	Intervention options confined to those tested in the trials; depends on achieving consensus	Not possible for “indivisible” interventions; composite intervention has not been tested in any of the trials	Not possible for “indivisible” interventions; depends on having sufficiently large dataset for meta-regression, and on the validity of the chosen theoretical mechanism
Effort	Minimal; consensus exercise and analysis	Several person-months of work	Several person-months of work

### Single-Trial-Based Choice—Choose among the Trial Interventions

Single-trial-based choice is essential when the intervention is considered “indivisible”, for example, because of the necessity for and/or interactions among components. For example, if each study used a different variation of a device or surgical implant, surgeons will need to select one implant rather than mix from the set of implants used in similar studies.

To choose from among the tested interventions, criteria are needed to make the basis for choice explicit. Such criteria may include the size and certainty of the estimates of the effect, the suspected or definite harms of the interventions, their applicability in particular settings, and their costs, acceptability, or practicality. Since no single intervention may be rated best on all these criteria, reviewers should ideally set out a tabulation of the choices, similar to the “buyer's guides” common in consumer magazines, that is perhaps best described as an intervention options table. Ideally the table should include the option of “no intervention”.

The apparent size of the effect requires particular consideration, as small studies with similar “true” effects will have greater dispersion, and hence some may appear to have larger effects by chance. Users should treat such small study effects with considerable caution. One potential method to reduce this problem is to provide shrinkage estimates [9] that combine the overall and individual estimates, with small studies receiving greater shrinkage than larger ones.

**Figure 1:**
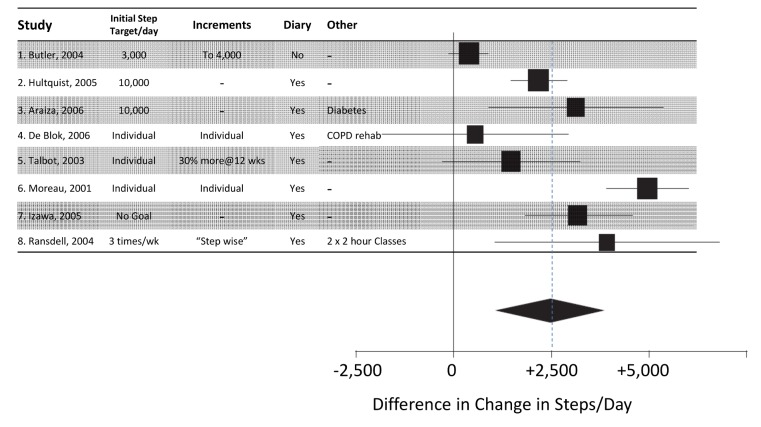
Trials of pedometer interventions to increase physical activity [18]: table of intervention elements of studies with forest plot of effect.

In selecting a single study, there will be less certainty about the effectiveness of the intervention, and sources of heterogeneity other than the intervention, such as population, setting, or methodology, should be considered. For example, consider the forest plot of studies assessing the effects of using pedometers to increase physical activity shown in Figure 1. Many users may be uncomfortable basing their intervention on trials 1 or 4 (in which the intervention apparently had a more modest effect than in the other studies, and did not yield statistically significant estimates of effect). Others may also not wish to use the interventions from studies 2 or 5 (as they yielded point estimates of effect that were less than the average estimate of effect, and the confidence interval in study 5 includes no difference). Further, if the interventions in all trials were similarly difficult to implement, but the interventions in studies 3, 6, and 8 were twice the cost of that in study 7, then the intervention from study 7 might be the preferred choice. However, the choice (and effect) may also vary with settings and populations, and if one study was performed in a population and setting most like that in which an intervention will be implemented, basing the intervention on this study may be the preferred choice.

A variant of the above process is to group the interventions that are sufficiently “similar” (across many possible dimensions) to be considered the same intervention. Provided this was specified a priori, the effect sizes might then be taken from a subgroup analysis based on these studies, rather than from the individual studies. An example is a systematic review of “autoinflation” for treating glue ear in children [10]: we asked an ENT surgeon, blind to the results of each study, to group together similar devices for autoinflation. These groups were then used for subgroup analysis (no differential effects were detected). This example also illustrates the need for a mix of expertise in the review group, including expertise in the disease and intervention domains.

### Common Components Hybrid—Recombination of Selected Components

If the interventions have multiple components, it may be possible to judge which components are likely to be necessary and/or effective and use these to propose a composite intervention. For example, Langhorne and Pollock [11] used all trials of specialized “stroke unit” care to identify the components judged to be most important, then surveyed the lead authors of those trials (who were involved in an individual patient data meta-analysis) to find out which components they planned to use and which they had actually used, hence deriving from their responses a proposed composite intervention. However, this composite intervention was a new intervention, which had not been formally tested; hence, caution is required in recommending and applying it. Ideally, the proposed composite intervention should be evaluated in a further trial of adequate statistical power.

Separation of the intervention into “components” is not straightforward. Depending on the type of intervention, the components may include the mode of delivery and materials, the intensity or dose used, the sequencing or scheduling of components, and so on. A number of checklists have been developed for different types of interventions to assist with this deconstruction [12],[13], and a generic checklist was recently developed [14], but further research in this area is needed.

After the deconstruction phase, the approach to the recombination of components will depend on how independent or dependent those components are and the quality and quantity of evidence of their effectiveness on their own, in combinations with each other, and with other intervention components. For example, many of the stroke unit trials included not only components of stroke management and measurement, but also education for staff in undertaking these components, and all three (staff education, measures, and management) may be needed for some elements to be effective.

If the interventions separate readily into multiple components believed to act independently of each other, then finding a composite intervention including these components is reasonable. The possible composite interventions range from those components common to all of the (effective) interventions, to a composite intervention that includes all of the components contained in any of the interventions assessed. For example, based on the systematic review of trials of stroke units described above, Langhorne and Pollock proceeded as follows. (i) They selected trials where the intervention was beneficial (in their method, the point estimate of the effect needed to favour the intervention, but did not need to be statistically significant). (ii) They identified key components from the included trials, protocols, and intervention manuals, then surveyed the authors of the trials selected to ask for additional components. (iii) They compiled the full list, then resurveyed the authors to ask which components they had actually used (preferably based on study data, but if that was not possible, then based on trial author's recall). (iv) They derived a composite intervention based on those components used in at least half of the trials in which the point estimate suggested a beneficial effect.

The above recombination process assumes that the more commonly used components are the most important ones, which may or may not be true. Clearly, it would be better to identify the minimal set of active components necessary to achieve any beneficial effects. One method of identifying the “active ingredients” of interventions [5] is to systematically specify the components of both the intervention and the control comparison conditions, using standardized taxonomies, and then use meta-regression to seek effects undetected by more conventional evidence synthesis methods [15],[16]. Sufficient numbers of studies and intervention data will not always be available or obtainable to allow this approach; hence, a pragmatic alternative is needed. Furthermore, if the initial set of studies is limited to those interventions that are apparently more effective, there is a loss of information on which to base subgroup analysis.

If there are sufficient independent components and sufficient trials (and intervention details), then several techniques, including subgroup analysis and/or meta-regression, may help to identify effective components. For example, Sherrington and colleagues [17] identified ten effective components of the interventions in 44 trials of exercise programmes to reduce falls. A meta-regression (which included quality and other non-intervention features in addition to the intervention features) found that programmes were more effective if they used a higher total dose of aerobic exercise and challenging balance exercises, and did not include a walking programme. That conclusion is helpful, but still requires some implementable specification of these effective components, as well as any common components.

Similarly, the systematic review (Figure 1) of pedometers (a multi-component intervention of which the device is only one part) included several subgroup analyses of different components of the interventions [18]. The authors concluded: “[H]aving a step goal was the key predictor of increased physical activity (P = .001). Indeed, there were no statistically significant improvements in physical activity in the 3 studies that did not include a step goal.” They also found that a step diary and non-workplace settings appeared to strengthen the effect, but there was no statistically significant effect associated with the brand of pedometer. However, this still leaves several different versions of the intervention (with step goals and diaries) to choose from.

For some interventions, the multiple components may simply be a collection of independent components with no dependence or interaction, such as balance exercises and home modification (floor repairs, grab rails, etc.) to prevent falls. However, when the components are dependent on or interact with one another, the composite methods outlined above may be neither feasible nor reasonable. As an illustration, imagine three trials of interventions to eradicate *Helicobacter pylori* infection using the same two antibiotics in combination but a different proton pump inhibitor in each—omeprazole, pantoprazole, or lansoprazole. Unless the reviewers recognized that these three “-azoles” were all drugs within the one class (proton pump inhibitors), rather than mistaking them as three different components, we might incorrectly conclude that, since antibiotics were the only component used in more than 50% of studies, the proton pump inhibitor was unnecessary. However, it may be possible to draw boundaries around some collections of components, and thereby create independent components again. This example suggests it may be important for the recombination process to be guided by an understanding of what role the components of an intervention play—that is, a theory predicting or explaining the intervention's action.

### Model-Guided Synthesis

A limitation of the common components hybrid approach is that we are restricted to the components used in the included trials. As the simplest example, suppose the drug doses used in the trials were all either 50 mg or 200 mg daily, and these appeared to have equivalent effects on the primary outcome. The common components hybrid approach would require using either 50 mg or 200 mg, but not 100 mg. However, pharmacological reasoning would suggest that 100 mg is likely to have an intermediate effect and but may have fewer or less troublesome adverse effects than the 200-mg dose. The analysis of a dose–response relationship is also possible for non-drug interventions, but requires that components of the interventions can be ranked by “dose”. For example, exercise for patients with heart failure, which improves symptoms and quality of life, would require an analysis of the duration or number of sessions, or the intensity of each. But this also requires some means of judging equivalence, such as achieving a particular percent of maximum heart rate. A dose–response analysis may help clarify which version of the intervention to use, and whether additional gains are worth the extra effort, cost, or side effects.

Assigning a “dose” requires identification of a central component (or components) of the intervention, and hence some understanding of its mechanism of action. Sometimes a dose–response analysis may be done directly by doing subgroup analyses on the different doses. However, statistically confident identification of differential effects of different doses requires a substantial amount of data in the strata being compared, and this may not be available.

**Figure 2:**
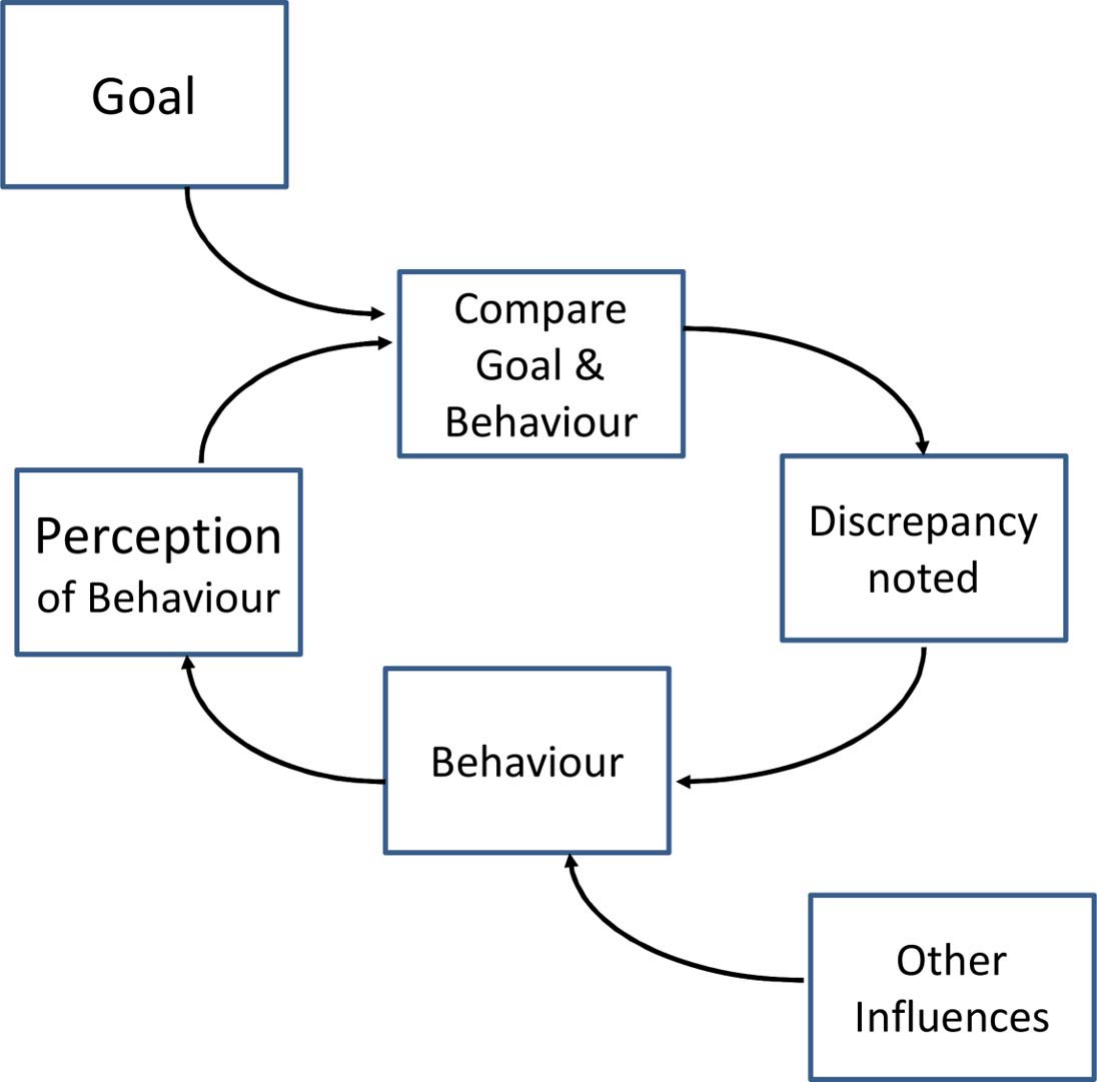
Example of the control theory model used for coding interventions in a review of audit and feedback studies by Gardner et al. [16].

More complex models of the mechanism of action might be used to guide the analysis of the components of the intervention. A model-guided analysis goes beyond recombination of components, and tries to identify the mechanism of action and the key components needed for the intervention to be effective. In a re-analysis of a Cochrane review [19] of audit and feedback for changing clinician behaviour, Gardner et al. [16] used control theory to analyse the effects of the interventions used. The control theory model (Figure 2) suggested that, to be effective, feedback should be accompanied by comparison with a behavioural target and by action plans. The authors coded all the trials to assess the extent to which the intervention incorporated these behaviour change techniques. The same analytic strategy was used in the updated Cochrane review [19], which had sufficient statistical power to test the theoretical prediction. A meta-regression based on the coded components of control theory found that interventions including a target and action plans were more effective than those including only feedback.

**Figure 3:**
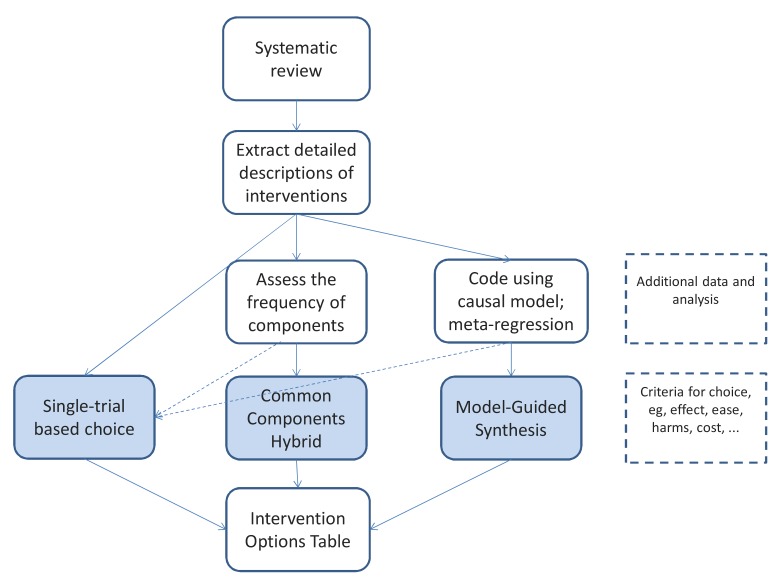
The steps from systematic review to a specific version of an intervention, showing the three basic approaches.

When this analysis of the model is complete, its confirmed elements may be used as a guide in selecting the most desirable version of the intervention (Figure 3). The precise details from the studies are still important as exemplars and options, but the theoretical understanding allows greater choice and adaptation.

A key limitation of the model-guided synthesis approach is the need for multiple trials with well-described interventions that provide sufficient variation and statistical power for subgroup analyses or meta-regression. Indeed, a danger is that an effective component is used by interventions in all trials, and hence the lack of variation between trials in this respect would mean that its effect would not be identified in the meta-regression. A further limitation is that meta-regression can be confounded by other study features, such as the population studied, the context, or the methods used [20], for which a meta-analysis based on individual patient data would be desirable but considerably increases the workload.

A supplementary approach to the model-analysis and meta-regression outlined above would be to also use an “accepted” surrogate outcome, rather than just the primary clinical outcome. For example, in an analysis of the effect of statins on cardiovascular disease, the authors showed not only that statins were effective overall, but also, using meta-regression [21], that the relationship between degree of cholesterol reduction and mortality reduction was approximately log-linear. The recommendation for intervention can thus be based on the marginal gain from increasing the degree of cholesterol reduction achieved by different drugs and doses. However, assumptions about the linearity of the dose–response relationship would need to be checked in each review.

## Discussion

Few systematic reviews currently provide much guidance on the specific forms of an intervention that should be used in different circumstances [7]. This has been partly due to a lack of awareness of the information needs of users of systematic reviews—clinicians, patients, policymakers—and partly a lack of explicit methods for analysing, synthesizing, and extrapolating from interventions grouped in the reviews. While the methods described in this article warrant further methodological development and testing, currently available methods should be more widely applied.

The choice of method for informing how best to translate review findings into evidence-based practice will depend on the types of interventions included, the nature of the data reviewed, and the resources available to reviewers. The appropriateness of different methods will depend on whether the interventions are “indivisible”, have single or multiple components, and can be ranked by intensity; whether and how the components interact; and the amount of data available for analysis. The composite methods for multi-component interventions require considerable extra work—Langhorne and Pollock's method required at least two rounds of surveys—which may not always be feasible or necessary. However, the simpler single-trial-based choice methods, particularly the presentation of an intervention options table, should always be possible, and can be done even if some of the interventions have incomplete or inadequate descriptions. In most cases, a method to guide the selection of an intervention could be reported in the review.

Given the different needs of different users in different countries, the intervention options table should provide a summary of the usable and feasible interventions in the review, with information on the criteria on which users would base their choice. Such a table could include both individual study interventions and synthesized interventions, providing users with a wider choice. Some interventions may also reasonably be omitted, for example, if they are insufficiently well described or contain inaccessible products. These decisions will require some judgement. For example, a recent NICE guideline for social anxiety disorder [22] recommended use of either of two “manualized” trial-based treatments, but did not recommend a synthetic approach because of uncertainties about interactions between elements [22].

There are several current barriers to applying these methods. The most problematic is inadequate description of interventions in the reports of primary studies [23]. Further work by authors, editors, and methodologists is required to improve the published descriptions available to reviewers and clinicians [6], in particular improved public access to protocols and protocol materials. However, we have demonstrated previously [8] that further details are often available from authors or other sources, and poor published descriptions cannot be used to justify ignoring this important aspect of systematic reviews [24].

Poor description applies not only to the content of interventions, but also to their mode of delivery, contextual features, and underlying theory. Several checklists have been developed to assist authors in publishing better descriptions of interventions, for example, for public health interventions [25], behavioural interventions [26], and non-pharmacological interventions more generally [13]. However, evaluations are of interventions that have been delivered and are most likely not the exact interventions that were planned. These checklists can be used to both specify planned and assess actual interventions. When this was done in relation to interventions to increase physical activity in those at risk of type 2 diabetes, it was found that 42% of the techniques specified in the intervention manual were delivered in practice [27]. This is a key issue for interpreting the results of systematic reviews since variation in adherence is likely to lead to variation in effect size [28].

In addition to the additional work involved, a central limitation of the common components hybrid and model-guided synthesis approaches is that the new “synthetic” composite intervention has not been tested formally in a controlled trial. If that leads to sufficient doubt about efficacy, then one way forward is to recommend a single-trial-based choice as the control group in a new comparison with the common components hybrid composite. Since there may be several uncertain components, researchers should consider factorial designs or phased adaptive designs [29].

Additional resources containing further details about interventions may support clinicians and policymakers in implementing the results of systematic reviews where detailed description of the intervention is lacking or where there is uncertainty based on heterogeneity within a category of included studies. For example, the Handbook of Non-Drug Interventions (HANDI; http://www.racgp.org.au/handi) aims to document details about non-pharmacological interventions to facilitate replication. Written by a panel of practitioners, with peer review from an expert in the intervention, resources such as this, and some clinical practice guidelines, may enable more formal approaches to choosing and describing an intervention from a systematic review.

While further work is warranted on all three of the basic approaches described here, the basic techniques are sufficiently clear for use in current systematic reviewing practice. As those doing systematic reviews will have done much of the work, we think they are in the best position to apply these methods, but recognize that the workload may be such that a separate report is required. However, as a minimum, those preparing systematic reviews could provide a table describing the elements of each version of the intervention studied. This table should also highlight differences between trials' interventions, to allow readers to judge more readily which might be most appropriate in their circumstances. If clinicians, patients, and policymakers are to be expected to apply the results of systematic reviews in practice, these approaches will need to be more widely adopted.
